# Breaking the Size‐Tolerance Trade‐off in Vertical Couplers via Adjoint‐Based Inverse Design

**DOI:** 10.1002/nap2.70211

**Published:** 2026-07-28

**Authors:** Ruiqi Liu, Suo Wang, Zeqiu Liu, Shiyu Mei, Jiahui Chi, Haiyang Yu, Yulian Cao, Jianguo Liu

**Affiliations:** ^1^ Nanometer Optoelectronics Lab Institute of Semiconductors, Chinese Academy of Sciences Beijing China; ^2^ College of Materials Science and Opto‐Electronic Technology University of Chinese Academy of Sciences Beijing China

**Keywords:** adjoint shape optimization, alignment and fabrication tolerance, heterogeneous integration, inverse design, vertical coupler

## Abstract

Heterogeneous integration of III–V lasers on silicon is pivotal for scaling next‐generation photonic systems, yet it faces a fundamental bottleneck: the inherent trade‐off between coupler compactness and misalignment tolerance. In this work, we break this longstanding barrier by combining multi‐start adjoint optimization with a physics‐guided strategy that places dense control points in near‐phase‐matched regions. This approach produces a more extended phase‐near‐matched region that spatially overlaps with the region of controlled odd supermode excitation, which is the key condition for simultaneously achieving compactness and tolerance. Our design achieves 98.11% coupling efficiency within a footprint of just 20 μm. Remarkably, it sustains over 80% efficiency under substantial misalignments up to ± 1.1 μm and waveguide width variations of ± 0.4 μm—representing the highest tolerance per unit length reported for vertical III–V‐on‐silicon couplers. This achievement decouples the traditional conflict between size and robustness, offering a critical solution for high‐yield micro‐transfer printing and dense photonic integration. The successful extension of our optimization framework to the lithium niobate‐on‐silicon nitride platform confirms its generality, positioning it as a versatile and transformative design strategy for a broad range of heterogeneous photonic platforms.

## Graphical abstract

A physics‐guided inverse design framework combines physical insight with adjoint optimization by placing dense control points in the phase‐near‐matched region. This guided strategy yields a 20 μm III–V‐on‐silicon coupler with 98.11% efficiency, > 80% efficiency at ± 1.1 μm misalignment, and record high tolerance per unit length.

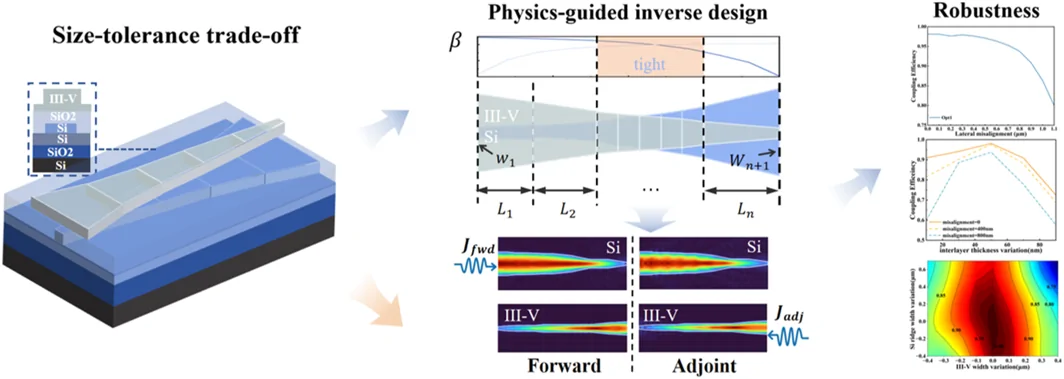

## 1 Introduction

Silicon photonics has established itself as a cornerstone technology for modern optical systems, driving advances in high‐speed communications [1], precision sensing [2], and low‐power computing [3]. Its dominance stems from the unparalleled scalability and cost‐effectiveness offered by CMOS‐compatible fabrication [4]. However, a critical bottleneck remains: Silicon is an indirect bandgap material, and this fundamental property makes it inherently inefficient at generating light [5]. To overcome this fundamental limitation, significant efforts have been devoted to integrating III–V gain media onto silicon, leading to strategies such as flip‐chip bonding [6, 7], hetero‐epitaxial growth [8, 9], and direct wafer bonding [10].

In recent years, micro‐transfer printing (μTP) has emerged as a particularly promising pathway to high‐performance silicon‐based lasers [11, 12]. μTP uniquely combines the benefits of using pre‐characterized III–V devices (as in flip‐chip) with the high‐throughput parallelism of wafer‐scale integration. This capability is indispensable for future large‐scale systems such as massive optical switch fabrics [13] and photonic neural networks [14], which demand the dense, co‐integration of thousands of laser elements on a single chip.

The realization of this ultra‐dense integration vision imposes exceptionally stringent requirements on the vertical optical coupler, the critical interface that transfers light between the printed III–V device and the underlying silicon waveguide. For μTP to be commercially viable, this coupler must be extremely compact to maximize component density, while simultaneously exhibiting exceptional tolerance to the inherent ± 1 μm‐scale placement inaccuracies of the printing process [15]. This creates a well‐known design paradox: traditional adiabatic couplers provide broad tolerance at the expense of sub‐millimeter‐scale lengths, which are prohibitive for dense integration [16, 17, 18, 19]; conversely, resonant couplers can be compact but are acutely sensitive to dimensional and alignment errors, making them incompatible with μTP's variability [20, 21, 22].

In response, a new class of hybrid couplers has been explored, aiming to combine the tolerance of adiabatic couplers with the compactness of resonant designs by deliberately relaxing perfect phase matching through multi‐segment tapers [23, 24]. These studies have shown that hybrid multi‐segment structures can provide an effective route toward balancing compactness and tolerance. However, an important challenge remains: how to systematically determine the geometric distribution that most effectively broadens the phase‐near‐matched region and aligns it with the coupling‐active region in a high‐dimensional and non‐intuitive design space. As the structural complexity increases, this task becomes increasingly difficult to accomplish through intuitive design alone.

To address this problem, we develop a physics‐guided inverse‐design framework. We combine a strategy that places dense control points in near‐phase‐matched regions with multi‐start adjoint optimization. In this way, physical insight guides where the design freedom is concentrated, whereas the algorithm efficiently searches for high‐performance solutions. Unlike population‐based algorithms [25, 26], the adjoint method needs only two simulations per iteration to calculate the gradient for all design variables at the same time [27, 28, 29]. Together with the multi‐start optimization scheme, we obtain an optimized structure with a more extended phase‐near‐matched region and a favorable spatial overlap with the controlled odd supermode excitation region. These two features together form the key condition for simultaneously achieving compactness and tolerance. Using this approach, we realize a 20 μm vertical coupler with 98.11% coupling efficiency and record tolerance per unit length for III–V‐on‐silicon vertical couplers. We further validate the generality of this framework by extending it to the lithium niobate‐on‐silicon nitride platform.

## 2 Device Design and Optimization Framework

Figure 1a illustrates the overall three‐dimensional structure and the x‐y cross‐section for our proposed III–V‐on‐silicon vertical coupler. This design is based on the typical III–V‐on‐silicon platform for optical amplifiers or lasers [30]. To simplify the analysis, we model the III–V region as a four‐layer stack: a p‐type InP (P‐InP) layer, a 250 nm thick *p*‐type separate confinement heterostructure (P‐SCH) layer made of AlGaInAs, an AlGaInAs multiple quantum well (MQW) layer comprising 8 wells, and an *n*‐type InP (N‐InP) layer. A 50 nm thick SiO_2_ bonding layer is introduced between the III–V stack and the Si waveguide, as commonly used in the μTP process. The lower Si rib waveguide has a thickness of 500 nm with an etch depth of 220 nm. It is worth noting that a 500 nm thick Si waveguide is chosen because its effective refractive index is close to that of the III–V waveguide. Moreover, this 500 nm SOI platform is supported by several foundries, such as Intel and UCSB.

**FIGURE 1:**
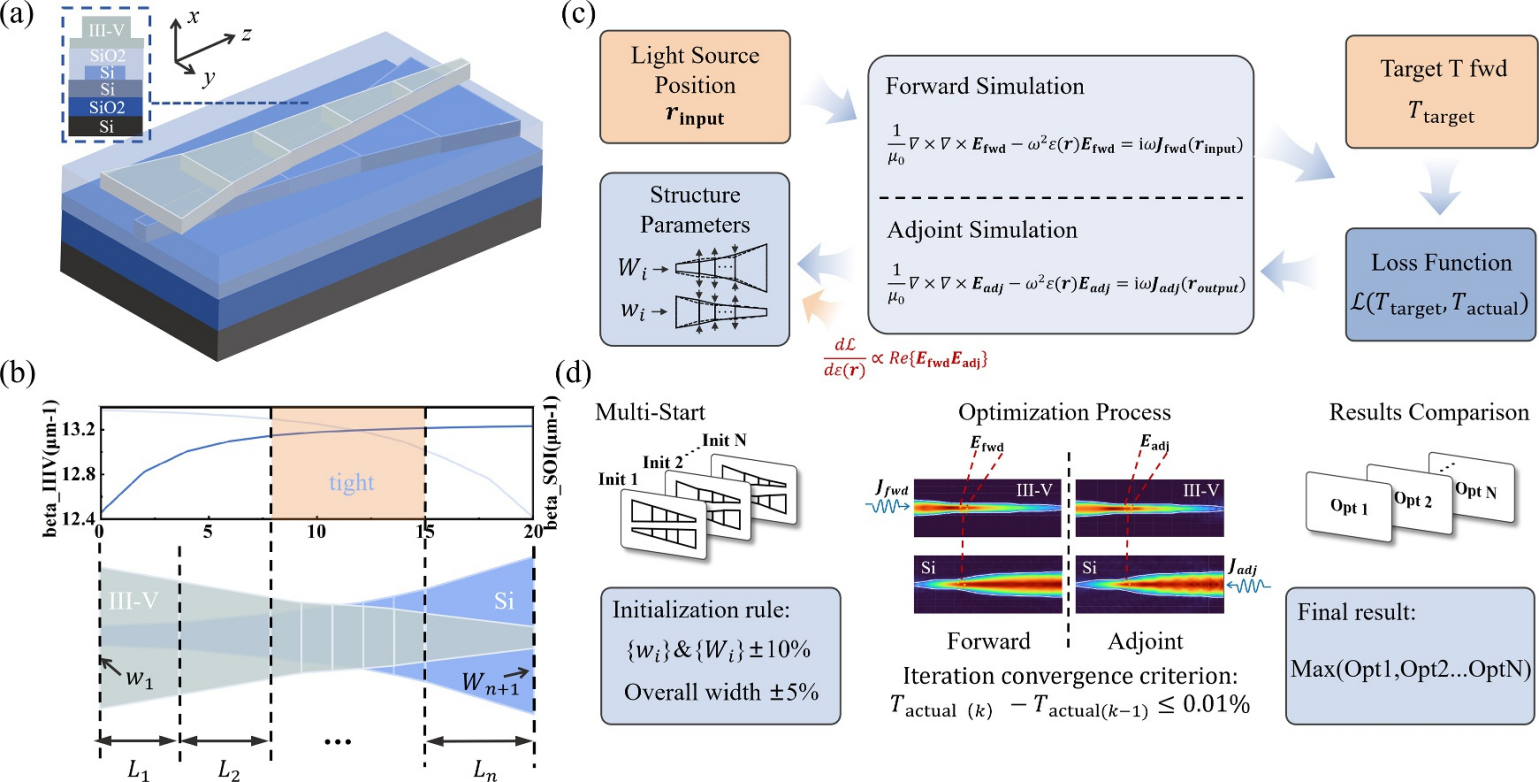
(a) 3D and cross‐section (*x*–*y*) illustrations of the vertical coupler, where the *x* and *y* axes define the transverse plane of the waveguide and the *z* axis represents the propagation direction. (b) Non‐uniform segment distribution in the coupler's *y*–*z* cross‐section, with increased segment density in regions where the β values of the III–V and Si waveguides closely match. (c) Design flow of the structure parameters of the vertical coupler via the Adjoint Shape Optimization Algorithm. (d) Multi‐start parallel optimization process, illustrating iteration convergence criterion and the selection of the optimal result from parallel outcomes.

As shown in Figure 1b, $W_{1}$/ $W_{n+1}$ and $w_{1}$/ $w_{n+1}$ are defined as the starting and ending width of the Si and III–V waveguide, respectively. Prior to entering this core coupling region, the III–V waveguide undergoes a preliminary “necking” stage, with its width reduced from the broad laser amplifier slab (e.g., ∼26 μm) to a moderate initial width (e.g., ∼4 μm) [31]. This width reduction does not significantly alter the optical mode, as the mode remains primarily confined within the III–V waveguide owing to its higher effective refractive index here. Within the main coupling section, because the III–V waveguide has a higher effective refractive index than the Si waveguide at the same width, the III–V width is gradually reduced to become smaller than the Si width, thereby achieving better phase matching. Specifically, $w_{1}$ and $w_{n+1}$ are set to 2.2 and 0.5 μm, whereas $W_{1}$ and $W_{n+1}$ are chosen as 0.4 and 3.5 μm, respectively, in order to achieve better phase matching in the middle section. Here, $w_{n+1}$ lies within the reported range of achievable widths for III–V waveguides [10, 12], and $w_{1}$ must exceed 2 μm to avoid hydrogen passivation, which would degrade the wall‐plug efficiency of the laser and consequently reduce its optical output power [31].

### 2.1 Principle of Tolerance Enhancement via Physics‐Guided Multi‐Segment Tapering

The performance of a vertical coupler can be fundamentally analyzed through coupled‐mode theory [32]. For a simplified case with constant coupling coefficient κ, the maximum power transfer p from one waveguide to the other is governed by:

(1) $$p=\frac{1}{1+\gamma^{2}},\text{where}\;\gamma \equiv \frac{\beta_{2}-\beta_{1}}{2\kappa },$$

here $\beta_{1}$ and $\beta_{2}$ are the propagation constants of the uncoupled III–V and silicon waveguides, respectively. Complete power transfer (p = 1) requires phase matching (γ=0), a condition that resonant couplers rely on but is highly sensitive to misalignment. The sensitivity stems from their operational principle: they are designed to precisely terminate at half the beating length $\left(L=\pi /\left(2\kappa \sqrt{1+\gamma^{2}}\right)\right)$ to capture the maximum power transfer. However, as lateral offset Δ*m* increases, the coupling coefficient *κ* decreases, causing *γ* to grow rapidly even if $\beta_{2}-\beta_{1}$ remains unchanged. Consequently, the optimal coupling length shifts dramatically, and any fixed‐length design no longer satisfies the mode‐beating condition for efficient power transfer. This leads to a sharp drop in efficiency, as described by *p* ∝ 1/(1 + *γ* ^2^).

As a first step, the limitations of a simple approach are illustrated in Figure 1b, which plots the simulated *β*‐profiles for the individual III–V and Si waveguide in a linear‐taper structure. This configuration produces a single, well‐defined point of perfect phase‐matching (where $\beta_{2}=\beta_{1}$). However, the phase mismatch $\left|\beta_{2}-\beta_{1}\right|$ grows rapidly on either side of this point. Critically, the linear taper geometry inherently lacks the degrees of freedom needed to engineer a broad, stable phase‐near‐matched region.

To actively engineer a broader and more robust region of low phase mismatch, we introduce a multi‐segment non‐uniform taper. The taper is conceptually divided into n segments, whose y‐z top‐view is aligned with the *β*‐profile plot in Figure 1b, visually demonstrating the design strategy. The multi‐segment taper is designed with two complementary roles: (i) in the central phase‐near‐matched region—precisely where the uncoupled *β* values naturally approach each other—the segment density is intentionally increased. This allocates more design degrees of freedom to the region where precise *γ*(*z*) control is critical for sustaining high efficiency under perturbation. (ii) Conversely, in the input and output sections where significant phase mismatch is inevitable, fewer segments are employed. The design problem then translates to optimizing the width profiles {$w_{i}$}(*i* = 2,…*n*)and {$W_{i}$} and length L, as will be detailed next.

### 2.2 Physics‐Guided Inverse Design With Adjoint Optimization

Based on the physical analysis in Section 2.1 and the non‐uniform multi‐segment design shown in Figure 1b, the design process for the vertical coupler is formulated as a physics‐guided constrained optimization problem, as depicted in the workflow of Figure 1c. The design process for the multi‐segment vertical coupler is formulated as a constrained optimization problem, as depicted in the workflow of Figure 1c. The objective is to minimize a loss function L that quantifies the deviation between the target power transmission $T_{\text{target}}$ and the simulated transmission $T_{\text{actual}}$, under specific physical and structural constraints. This is expressed as:

$$\text{Minimize}\;L\left(T_{\text{target}},T_{\text{actual}}\left(p\right)\right)$$

$$\text{Subject}\;\text{to}\;\theta \left(r\right)<\frac{\lambda }{2W\left(r\right)\cdot n_{\text{eff}}\left(r\right)}$$

(2) $$\frac{1}{\mu_{0}}\nabla \times \nabla \times E_{\text{fwd}}-\omega^{2}\varepsilon \left(r\right)E_{\text{fwd}}=i\omega J_{\text{fwd}}\left(r_{\text{input}}\right),$$

here $p=\left\{w_{i},W_{i}\right\}$ denotes the trainable width parameters of the silicon and III–V waveguide segments. The first constraint is a criterion for suppressing higher‐order modes. It ensures the local taper angle θ(r) remains below the fundamental mode's diffraction limit, thereby preventing energy leakage into unwanted higher‐order modes [33]. The second constraint enforces Maxwell's equations for all simulation cases. The electromagnetic fields are solved numerically using the finite difference time domain (FDTD) method.

To solve this problem efficiently, we employ a gradient‐based adjoint optimization framework. As outlined in Figure 1c, each iteration only consists of a forward and an adjoint simulation. The forward simulation computes the electric field $E_{\text{fwd}}$ from the III–V waveguide for a given input source $J_{\text{fwd}}\left(r_{\text{input}}\right)$, from which the loss function L is evaluated. The adjoint simulation then computes the gradient $\nabla_{p}L$ for all design parameters simultaneously, by evaluating ∂L/∂ε and projecting it onto the parameter space. Once the gradient is obtained, a shape variation Δp is proposed [29]. This update is then projected onto the feasible design space defined by the higher‐order mode suppression constraint.

As indicated in Figure 1d, we employ a multi‐start optimization strategy to mitigate the risk of converging to suboptimal local minima—a common challenge in non‐convex photonic design problems. Multiple independent optimization runs(labeled as Opt1,Opt2,…,OptN) are executed in parallel, each initialized with a distinct parameter set *p* ^(0)^. Specifically, all runs are initialized by randomly perturbing a simple linear taper: apart from the fixed start/end widths ($w_{1},w_{n+1},W_{1},W_{n+1}$), all intermediate widths are perturbed within ± 10%, followed by a global width scaling within ± 5%. Each optimization run proceeds iteratively using our gradient‐based adjoint method. The process terminates when the change in simulated transmittance between consecutive iterations is negligible, that is, $T_{\text{actual}\;\left(k\right)}-T_{\text{actual}\;\left(k-1\right)}$ ≤ 0.01%. The final design is then selected from the run achieving the lowest loss. This strategy enhances the robustness of the design process.

## 3 Results and Discusssion

### 3.1 Optimization Process Analysis and Design Selection

Figure 2a illustrates the convergence behavior of a typical optimization run (Opt1). Utilizing our gradient‐based adjoint method, the coupler's simulated coupling efficiency rapidly increases with iteration count. The optimization process terminates when the change in efficiency between consecutive iterations falls below the predefined threshold of 0.01% (i.e., $T_{\text{actual}\left(k\right)}-T_{\text{actual}\left(k-1\right)}$ ≤ 0.01%). As shown, Opt1 converges to a peak efficiency of 98.11% after only 29 iterations, demonstrating the remarkable speed and precision of our adjoint optimization framework.

**FIGURE 2:**
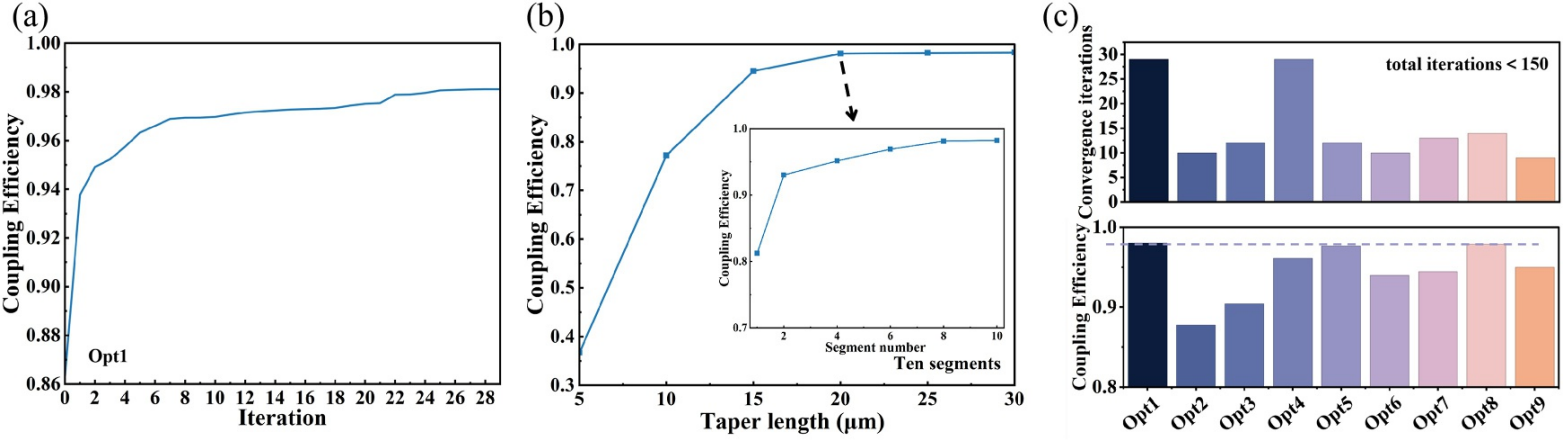
(a) Iteration process of Opt1 showing the evolution of coupling efficiency. (b) Simulated coupling efficiency of the optimized couplers versus lengths. The insert shows the coupling efficiency of 20 μm couplers with different numbers of taper segments. (c) Coupling efficiency versus the required number of iterations for nine parallel optimization processes.

The determination of the optimal coupler length and segmentation strategy is presented in Figure 2b. We investigate the relationship between coupling efficiency and total taper length for a structure with 10 non‐uniform segments. The efficiency increases with length and begins to saturate at approximately 20 μm, reaching near the theoretical maximum of 98.11%. Extending the length beyond this point yields diminishing returns in performance gain while linearly increasing the footprint. Therefore, 20 μm is selected as the optimal length, achieving an exceptional balance between compactness and high efficiency. The inset of Figure 2b further explores the impact of the number of taper segments at this fixed 20 μm length. The coupling efficiency improves significantly as the segment count increases from 1 (a simple linear taper) to 10, but the enhancement rate diminishes beyond 10 segments, indicating performance saturation. This justifies our choice of a 10‐segment, non‐uniform structure, which provides sufficient degrees of freedom for precise phase management without unnecessary optimization complexity.

Figure 2c validates the effectiveness and computational efficiency of our multi‐start parallel optimization strategy. Nine independent optimization runs are executed concurrently, each initialized with a distinct parameter set as described in Section 2.2. The final coupling efficiencies of all nine runs are plotted against their total required iterations. Among them, three runs—Opt1 (98.11%), Opt5 (97.63%), and Opt8 (97.90%)—achieve notably higher efficiencies, emerging as the leading candidates. Crucially, all runs converge in under 150 iterations total, with the best‐performing ones requiring fewer than 30 iterations each.

A comparative analysis of the leading candidates (Opt1, Opt5, and Opt8) shows that all three designs achieve high nominal coupling efficiency, but their internal mode evolution is different. Here, the phase‐near‐matched region is identified using a practical criterion that the relative mismatch $|\beta_{\text{III}-V}-\beta_{\text{Si}}|/\beta_{\text{avg}}$ ≤ 5%, where $\beta_{\text{avg}}$ denotes the average of $\beta_{\text{III}-V}$ and $\beta_{\text{Si}}$. Among the three candidates, Opt1 shows the broadest low‐mismatch region. It also shows the best overlap between this region and the odd supermode excitation region. As discussed in Section 2.1, this overlap is important for achieving both compactness and robustness. Opt5 and Opt8 also exhibit locally phase‐near‐matched regions, but these regions are shorter or less well aligned with the odd supermode excitation region. Detailed comparisons of the modal evolution, phase‐matching behavior, and field distributions of these candidates are provided in Supporting Information S1: Figure S1. Based on this combined consideration of efficiency and robustness, Opt1 is selected as the final design.

Figure 3 presents the modal evolution and field transfer characteristics of the selected design, Opt1. More importantly, it illustrates the central idea of this work: physical guidance and adjoint optimization act together to shape the coupling process. The physical guidance places more design freedom in the phase‐critical region, whereas the optimization determines how this freedom should be used to obtain the desired mode evolution. To quantify the excitation of even and odd supermodes in our structure, we decompose the input field into the two supermodes using the method described in the Supporting Information S1: Section A. The corresponding power fractions $T_{\text{eve}n}$ and $T_{\text{odd}}$ are calculated via Supporting Information S1: Equations (S1–S4).” At the input section, the even supermode is concentrated in the III–V waveguide and therefore dominates the initial excitation, As shown in Figure 3a, the optical power is initially concentrated mainly in the even supermode. As propagation proceeds, a controlled portion of the odd supermode is excited in the central section of the coupler. This indicates the intended hybrid coupling behavior rather than a strictly adiabatic transfer process. The corresponding evolution of the propagation constants of the uncoupled III–V and Si waveguides is shown in Figure 3b. Using the practical criterion described above, the phase‐near‐matched region of Opt1 extends from about 7 to 13 μm. More importantly, the odd supermode fraction remains above 20% throughout this interval. This means that the low‐mismatch region and the coupling‐active region overlap well. This overlap is the direct result of the combined action of physical guidance and optimization, and it is the key reason why Opt1 can achieve both compact size and high tolerance. Figure 3c further shows that, at the output plane (*z* = 20 μm), the optical field has been transferred predominantly from the III–V waveguide to the underlying Si waveguide, with only a small residual field remaining in the III–V region.

**FIGURE 3:**
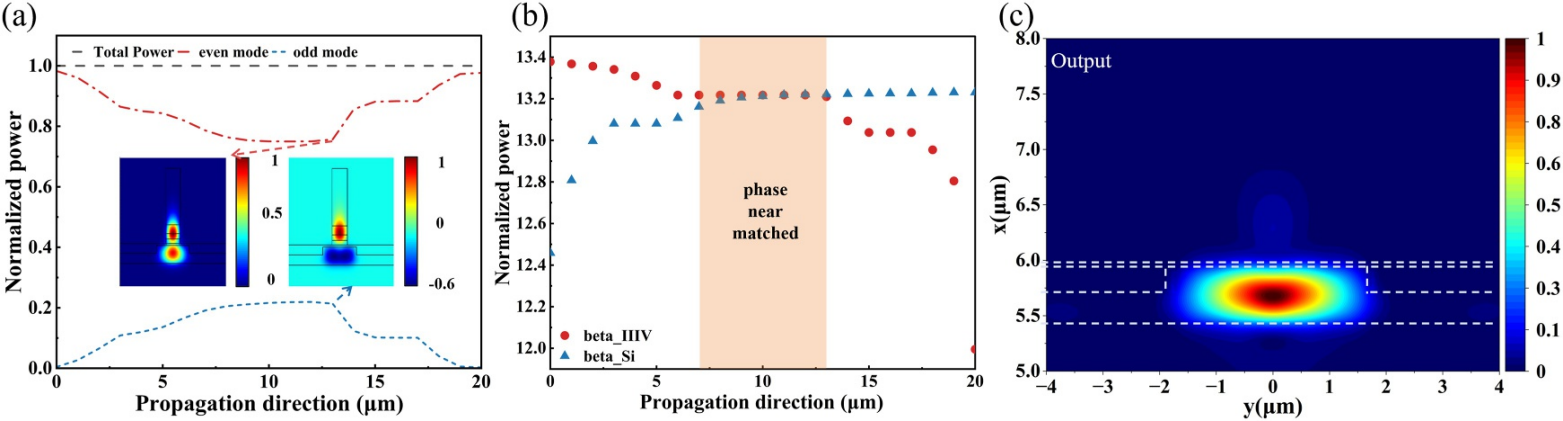
(a) Normalized power of the even and odd supermodes along the propagation direction for the optimized coupler in Opt1. Inserts show the simulated transverse electric field distribution at *z* = 13 μm. (b) Simulated β distribution for III–V waveguide and Si waveguide along the propagation direction. (c) Simulated electric field distribution at *z* = 20 μm cross section for the optimized vertical coupler.

### 3.2 Alignment and Fabrication Tolerance of the Optimized Coupler

The robustness of the selected optimum (Opt1) is quantitatively evaluated in Figure 4. Figure 4a shows the simulated coupling efficiency as a function of lateral misalignment (Δ*m*). Under perfect alignment, the coupler achieves 98.11% efficiency. More importantly, it maintains a high efficiency of > 80% across a misalignment range of ± 1.1 μm, demonstrating excellent alignment tolerance compatible with μTP integration requirements. This excellent tolerance to lateral misalignment can be attributed to the optimized geometry of the multi‐segmented coupler. As indicated by the mode dynamics in Figure 3, the intentionally broadened phase‐near‐matched region and its alignment with the mode‐beating‐assisted coupling section ensure that even under lateral offset—which primarily reduces the coupling coefficient *κ*—the condition for effective power transfer (|*γ*| ≈ 0) can still be satisfied over a substantial portion of the coupler. Therefore, the coupling efficiency remains high across a wide range of Δ*m*.

**FIGURE 4:**
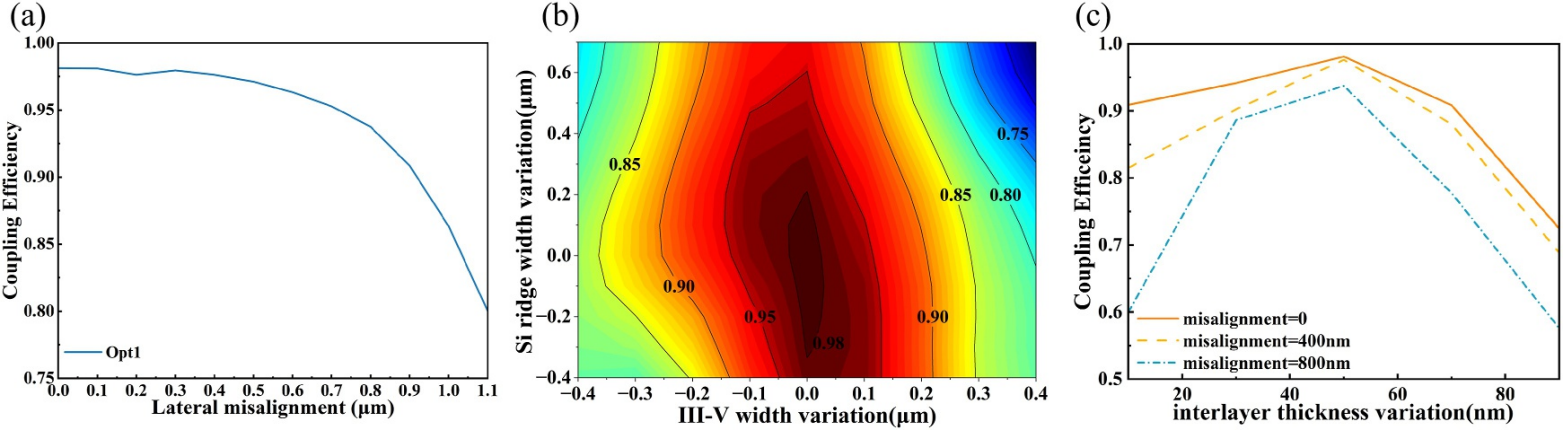
(a) Simulated coupling efficiency versus the lateral misalignment for the optimized couplers in Opt1. (b) Simulated map of the coupling efficiency with different width variation of the III–V and Si waveguide. (c) Simulated coupling efficiency versus interlayer thickness under different lateral misalignments.

Figure 4b presents a simulated map of the coupling efficiency under simultaneous width variations of both the III–V and Si waveguides. The coupler exhibits remarkable width tolerance, which stems directly from its non‐uniform multi‐segment design. Any uniform deviation in waveguide width effectively shifts the position of the phase‐matched point along the propagation direction *z*. Due to the intentionally extended and gently tapered central region (see Figure 3b), a low‐mismatch condition can still be maintained around this shifted point, thereby preserving high coupling efficiency. Quantitatively, when the coupler is perfectly aligned, the tolerable width variation range (> 90%) reaches −0.4 to + 0.7 μm for the Si waveguide and (> 80%) −0.4 to + 0.4 μm for the III–V waveguide. The efficiency remains above 80% when both waveguides vary simultaneously within ± 0.3 μm.

Figure 4c investigates the tolerance to variations in the SiO_2_ interlayer thickness, which is critical because the bonding layer thickness can fluctuate by at least ± 10 nm in practical μTP or bonding processes. Although the designed thickness of the SiO_2_ layer is 50 nm, the coupling efficiency keeps higher than 80% with the thickness varying from 0 to 80 nm when 0.4 μm misaligned or varying from 25 to 70 nm when 0.8 μm misaligned.The design maintains > 90% efficiency for thickness variations from 0 to 70 nm under perfect alignment, demonstrating a robust process window that accommodates typical fabrication variations.

The superiority of our physics‐guided design is further supported by a direct comparison with a uniformly segmented coupler optimized under the same adjoint framework but blindly initialized. The detailed results are provided in the Supporting Information S1: Sections C and D. In brief, although both designs achieve similarly high nominal efficiency, the uniformly segmented coupler exhibits a relative phase mismatch that is consistently three times larger within the critical coupling region, which directly leads to weaker tolerance under alignment and fabrication variations.

The comparison confirms that our strategy—concentrating design freedom in the near‐phase‐matched region guided by physical insight—is the key to achieving both high efficiency and record robustness. By placing dense control points in this sensitive region, our physics‐guided approach naturally produces a width evolution curve with a broad flat region where the relative mismatch stays below 0.88% over 9–13 μm and the odd supermode fraction exceeds 20%. This favorable geometry is a direct result of the physics‐informed inverse design strategy.

## 4 Extended Application of the Proposed Algorithm

The broad applicability of our design framework is demonstrated by its successful application to the heterogeneous LiNbO_3_(LN)‐on‐Si_3_N_4_ platform—a promising system that combines LN's strong Pockels effect [34, 35] with Si_3_N_4_'s low loss for high‐performance modulators [36, 37]. This platform faces the same fundamental compactness‐tolerance trade‐off in vertical coupling, which our method directly addresses.

We design and evaluate a coupler using our framework (multi‐initialization adjoint optimization guided by non‐uniform segmentation). Compared to a 45 μm reference design with a fixed‐width Si_3_N_4_ waveguide [38], our optimized structure is only 25 μm long and features tapers in both waveguides: the Si_3_N_4_ width reduces from 3 to 0.3 μm, whereas the LN varies from 0.4 μm(a practical lower limit) to 3.5 μm for improved phase matching.

As comprehensively validated in Figure 5, the coupler achieves 92% efficiency with rapid algorithm convergence (Figure 5a). The slightly lower efficiency (compared to > 98% for III–V/Si) stems directly from the weaker optical confinement in the low‐index‐contrast LN‐on‐Si_3_N_4_ system, where mode leakage into the cladding and substrate introduces an intrinsic loss mechanism. Our peak efficiency is marginally below the 94.4% reported in Ref. [38], as we intentionally use a wider LN tip width (400 nm) to reduce sidewall scattering loss. Notably, that work reports a much lower experimental efficiency of only 53.7%, attributing it primarily to sensitivity to tip‐width variation and alignment. This result highlights the practical value of our design philosophy.

**FIGURE 5:**
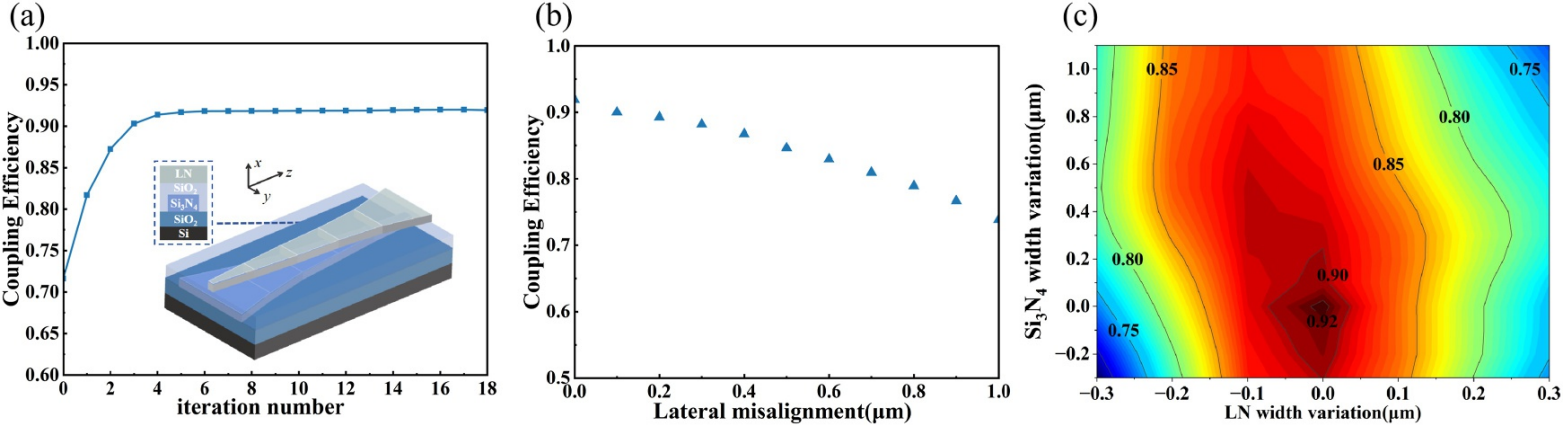
(a) Iteration process showing the evolution of coupling efficiency. (b) Simulated coupling efficiency versus the lateral misalignment. (c) Simulated map of the coupling efficiency with different width variation of the LN and Si3N4 waveguide.

Consistent with this philosophy, our design demonstrates exceptional simulated tolerance: it maintains > 80% efficiency under a ± 0.7 μm misalignment (Figure 5b) and keeps > 80% efficiency across LN waveguide width variations of ± 0.2 μm (Figure 5c), significantly outperforming reference designs. These results affirm that our framework translates its core principles into fabrication‐robust, high‐performance couplers across materially distinct platforms.

The final performance metrics of our optimized III–V‐on‐silicon coupler are benchmarked against prior art in Table 1. Our design achieves 98.11% coupling efficiency at an ultra‐short length of only 20 μm, along with ± 1.1 μm alignment tolerance and ± 0.4 μm III–V width tolerance. To quantitatively compare the trade‐off between performance and footprint, we introduce two normalized figures of merit: FOM_1_ = (Alignment Tolerance Range)/Length and FOM_2_ = (Width Tolerance Range)/Length. As summarized in Table 1, our coupler demonstrates record‐high values for both FOM_1_ (0.11 μm^−1^) and FOM_2_ (0.04 μm^−1^), significantly outperforming all previously reported designs for III–V‐on‐silicon vertical couplers. Furthermore, when extended to the LN‐on‐Si_3_N_4_ platform, the same design methodology yields a compact 25 μm coupler with 92% efficiency, ± 0.7 μm alignment tolerance, and corresponding FOM values that maintain state‐of‐the‐art tolerance‐per‐length performance.

**TABLE 1:** Comparison of the performance of different vertical couplers.

References	Principle	Materials	Length (μm)	Efficiency (%)	FOM1^a^	FOM2^b^
[17]	Adiabatic	Si_3_N_4_/Si	500	97	1.1^c^	N.A.
[18]	Adiabatic	AlGaInAs/Si	250	100	1.28^c^	N.A.
[19]	Adiabatic	InGaAsP/InP	175	94	N.A.	N.A.
[38]	Adiabatic	LN/Si_3_N_4_	45	94^c^	1.92^c^	N.A.
[21]	Resonant	InGaAsP/InP	24	98	N.A.	1.25^c^
[22]	Resonant	AlGaInAs/Si	8	95	2.5^c^	N.A.
[23]	Hybrid	AlGaInAs/InP	55	96	1.82^c^	1.02^c^
[24]	Hybrid	AlGaInAs/Si	87	98	2.52^c^	1.15
This work	Hybrid	AlGaInAs/Si	20	98	11	4
This work	Hybrid	LN/Si_3_N_4_	25	92	5.6	1.6

^a^ Misalignment tolerance/length(coupling efficiency ≥ 80%).

^b^ Width variation tolerance/length(coupling efficiency ≥ 80%).

^c^ Data are obtained from figures in the references.

## 5 Conclusions

This work addresses the critical need for compact and highly tolerant vertical couplers in advanced heterogeneous integration technologies, such as micro‐transfer printing. We propose a physics‐guided inverse‐design framework that combines physics‐guided non‐uniform segmentation with a multi‐initialization adjoint algorithm. The innovation is how physical insight guides gradient‐based optimization: the non‐uniform segmentation strategically concentrates design degrees of freedom in the phase‐near‐matched region, as informed by coupled‐mode theory, to flatten the effective phase‐mismatch landscape. The multi‐initialization adjoint method efficiently navigates the high‐dimensional design space, mitigating the risk of local minima and ensuring robust convergence.

Applying this framework to a III–V‐on‐silicon platform, we design an ultra‐compact vertical coupler with a total length of only 20 μm. The optimized device achieves 98.11% coupling efficiency within a 20 μm footprint, while sustaining over 80% efficiency under ± 1.1 μm misalignment and ± 0.4 μm width variation. To our knowledge, the resulting figures of merit—FOM_1_ = 11 μm^−1^ (alignment tolerance per unit length) and FOM_2_ = 4 μm^−1^ (width tolerance per unit length)—are the highest reported for III–V‐on‐silicon vertical couplers. This performance decouples the traditional size–robustness trade‐off, offering a critical solution for high‐yield micro‐transfer printing and dense photonic integration.

We further validate the generalizability of our design methodology by successfully applying it to a LN‐on‐Si_3_N_4_ platform. The resulting 25 μm‐long coupler shows markedly improved alignment tolerance compared to a reference design, maintaining over 80% efficiency at a ± 0.7 μm misalignment. This extension confirms that our framework is a versatile tool capable of addressing coupling challenges across diverse material systems.

In conclusion, this study provides a highly efficient and robust design strategy for vertical couplers, directly tackling the integration bottlenecks in silicon photonics and beyond. The demonstrated coupler sets a new state‐of‐the‐art in balancing miniaturization and fabrication tolerance. By offering a generalizable pathway to design high‐performance couplers for various heterogeneous platforms, this work contributes significantly to the development of future large‐scale, multi‐functional, and manufacturable photonic integrated circuits.

## Author Contributions

**Ruiqi Liu:** conceptualization, methodology, writing – review and editing, writing – original draft, data curation, software, investigation, validation, formal analysis, visualization, resources. **Suo Wang:** writing – review and editing, funding acquisition, methodology, supervision, writing – original draft. **Zeqiu Liu:** methodology. **Shiyu Mei:** validation, data curation. **Jiahui Chi:** validation. **Haiyang Yu:** project administration, funding acquisition. **Yulian Cao:** writing – review and editing, supervision. **Jianguo Liu:** supervision, funding acquisition.

## Funding

The study was supported by the National Natural Science Foundation of China (62405308), the Beijing Natural Science Foundation (4262091), Group Notice Young Elite Scientists Sponsorship Program of the Beijing High Innovation Plan (20250648).

## Conflicts of Interest

The authors declare no conflicts of interest.

## Supporting information


Supporting Information S1

## Data Availability

The data that support the findings of this study are available from the corresponding author upon reasonable request.
