# Supplementary material for: Breaking the Size‐Tolerance Trade‐off in Vertical Couplers via Adjoint‐Based Inverse Design
Source: Nanophotonics. 2026 Jul 28;15(15):e70211. doi: 10.1002/nap2.70211 (PMC13642114; doi:10.1002/nap2.70211)
Supplement: Supplementary file 1 [file NAP2-15-e70211-s001.docx]

supplemental document

A. **Caculation of the even and odd supermode fractions**

Following the method in Ref. [S1] under the non-absorbing and bound-modes approximation, the complex transmission coefficient of the forward propagating waves can be written as:

$$a_{j}=0.25*\left( \left. \frac{\int d\boldsymbol{A}\cdot\boldsymbol{E}_{in}\times\boldsymbol{H}_{j}^{*}}{N_{j}}+\frac{\int d\boldsymbol{A}\cdot\boldsymbol{E}_{j}^{*}\times\boldsymbol{H}_{in}}{N_{j}^{*}} \right) \right.$$

(S1)

Here, the modal normalization constant $N_{j}$ is defined as:

| $N_{j}$=$0.5*\int d\boldsymbol{A}$·$\boldsymbol{E}_{j}\times\boldsymbol{H}_{j}^{*}$ | (S2) |
| --- | --- |

The total input power on a certain plane is defined as:

| $P_{in}$*=*$0.5*\int d\boldsymbol{A}$*·*$\boldsymbol{E}_{in}\times\boldsymbol{H}_{in}^{*}$ | (S3) |
| --- | --- |

The power fraction of mode $j$ is then calculated as:

$$T_{j}=\frac{{|a_{j}|}^{2}*|N_{j}|}{P_{in}}$$

(S4)

Here, $\boldsymbol{E}_{in}$ and $\boldsymbol{H}_{in}$ are the electric and magnetic fields of the input field, $\boldsymbol{E}_{j}$ and $\boldsymbol{H}_{j}$ are the eigenmode fields of mode $j$, $d\boldsymbol{A}$ is the surface normal vector of the integration cross-section.

B. **Selection of the Optimal Physics-Guided Non-Uniformly Segmented Coupler**

To identify the best design from the candidates Opt1, Opt5, and Opt8, we compare the modal evolution, phase-matching behavior, and output field distributions of the three leading candidates, Opt1, Opt5, and Opt8, in Fig. S1. The same practical criterion used in the main text is adopted here, namely that the phase-near-matched region is identified by a relative mismatch $|\beta_{III-V}-\beta_{Si}|/\beta_{\mathrm{avg}}$≤ 5‰.

Figure S1(a)-(c) shows that the three candidates exhibit different odd supermode evolution, although all of them achieve high nominal coupling efficiency. For Opt1, the odd supermode is excited in the central part of the coupler, where the main power transfer is intended to occur. By contrast, for Opt5 and Opt8, the odd supermode excitation shifts more toward the later part of the device, indicating a less favorable coupling process.

This difference is consistent with the phase-matching behavior in Fig. S1(d)-(f). For Opt1, the phase-near-matched region extends from about 7 to 13 μm, and the odd supermode fraction remains above 20% throughout this interval. By comparison, the phase-near-matched regions of Opt5 and Opt8 are both noticeably shorter than that of Opt1. In addition, the odd supermode fraction remains below 20% within these shorter low-mismatch intervals. This indicates a weaker overlap between the phase-near-matched region and the coupling-active region.

The field distributions in Fig. S1(g)-(i) support the same trend. Opt1 shows a cleaner transfer of optical power from the III-V waveguide to the Si waveguide at the output. By contrast, Opt5 and Opt8 show more residual field remaining in the III-V waveguide, indicating a less effective transfer process.

From this comparison, Opt1 is chosen as the representative non-uniformly segmented coupler for the main text and for the following comparisons.

Fig.S1. Comparison of the modal evolution, phase-matching behavior, and output field distributions of three representative non-uniformly segmented couplers obtained from the multi-start optimization: Opt1, Opt5, and Opt8. (a)-(c) Normalized power of the even and odd supermodes along the propagation direction for the optimized couplers in Opt1, Opt5 and Opt8. (d)-(f) Simulated $\text{β}$ distribution for III-V waveguide and Si waveguide along the propagation direction( Opt1,Opt5 and Opt8). (g)-(i) Simulated electric field distribution at *y*=0 cross section for the optimized vertical coupler( Opt1,Opt5 and Opt8).

C. **Mechanistic Advantage of the Optimal Non-uniformly Segmented Coupler over a Uniformly Segmented, Blindly Initialized Coupler**

To clarify the physical advantage of our physics-guided strategy, we compare the selected non-uniformly segmented coupler with a uniformly segmented coupler which is blindly initialized. Both structures are optimized within the same adjoint-based framework, but only the non-uniform design intentionally concentrates geometric degrees of freedom in the near-phase-matched region.

Fig.S2 Comparison of the selected non-uniformly segmented coupler and the optimized uniformly segmented coupler(a),(c) Normalized power of the even and odd supermodes along the propagation direction for the optimized non-uniform and uniform couplers. (d),(f) Simulated $\text{β}$ distribution for III-V waveguide and Si waveguide along the propagation direction for the optimized non-uniform and uniform couplers. (b),(e) The detailed $\text{β}$ distribution for III-V waveguide and Si waveguide along the propagation direction in the phase-near-matched region.

In figure S2, we compare the modal evolution and phase-matching behavior of the two couplers. As is concluded in Section A,for the optimal non-uniform coupler, the phase-near-matched region (7 - 13 μm) maintains an odd supermode fraction above 20%. The uniformly segmented coupler exhibits a slightly shorter such region (8-13 μm) with a similarly high odd supermode fraction [S2(c),(f)]. The critical difference lies in the quality of phase matching [S2(b),(d)]: within the near-matched zone, the non-uniform design suppresses the relative mismatch below 0.88‰ across a broad range (9-13  μm). In contrast, the uniform design consistently shows a mismatch that is three times larger or more.

This difference arises from where the control points are placed. In the non-uniform design, dense control points are concentrated in the central region - where the uncoupled propagation constants naturally approach each other and the structure is most sensitive to geometric changes. This allocation gives the optimizer the freedom to finely shape the width profile, resulting in a broad flat region (9-13 μm) where the relative mismatch stays below 0.88‰ [Fig. S2(b)]. Importantly, this low-mismatch flat region aligns with the area where the odd supermode fraction exceeds 20% [Fig. S2(c)], ensuring efficient power transfer under the most favorable phase condition. The uniformly segmented coupler, by contrast, distributes control points evenly. With fewer degrees of freedom in the sensitive central region, the optimizer cannot achieve the same precision. The resulting low-mismatch zone is narrower and less flat [Fig. S2(e)]. Consequently, the phase mismatch remains larger, and the device is more sensitive to perturbations.

D. **Tolerance Comparison Between the Optimal Non-uniformly and Uniformly Segmented Couplers**

Figure S3 compares the tolerance performance of the optimized non-uniformly segmented coupler and the uniformly segmented coupler. Under perfect alignment, the two designs show similar coupling efficiency, with 98.11% for the non-uniformly segmented coupler and 97.65% for the uniformly segmented coupler.

Fig.S3 Tolerance comparison between non-uniformly segmented coupler and uniformly segmented coupler. (a) Simulated coupling efficiency versus the lateral misalignment for the two couplers. (b) Simulated coupling efficiency versus interlayer thickness. (c) Simulated coupling efficiency with different width variation of the III-V waveguide.

A clearer difference appears once perturbations are introduced. As shown in Fig. S3(a), misalignment of 0.9 μm, whereas the efficiency of the uniformly segmented coupler decreases to 79.5% under the same condition. Figure S3(b) shows a similar trend for bonding-layer-thickness variation. For the uniformly segmented coupler, a thickness deviation of 20 nm already causes obvious degradation, and the coupling efficiency drops to below 80% when the SiO_2_ thickness is reduced to 30 nm. Figure S3(c) shows the fabrication-tolerance comparison. Under a III-V waveguide width variation of ±0.3 μm, the coupling efficiency of the uniformly segmented coupler decreases to 76.26% and 80.54%, respectively.

These results are consistent with the phase-matching behavior shown in Fig. S2. The non-uniformly segmented coupler maintains a broader low-mismatch region and a better overlap with the coupling-active region, so the coupling process is less sensitive to alignment and fabrication variations.

References

[S1] A. W. Snyder and J. D. Love. Optical Waveguide Theory. London: Kluwer Academic Publishers (1983).
